# Cross-kingdom RNA in plant–microbe interactions: from molecular interactions to next-generation crop protection

**DOI:** 10.1007/s44154-026-00337-x

**Published:** 2026-09-23

**Authors:** Borala Liyanage D. Deepali, Caiping Huang, Wei Zhang, Hui Wang, Yuan Wang, Kevin D. Hyde, K. W. Thilini Chethana, Jiye Yan

**Affiliations:** 1 Key Laboratory of Environment Friendly Management On Fruit Diseases and Pests in North China, Institute of Plant Protection, Beijing Academy of Agriculture and Forestry Sciences, Beijing, 100097 China; 2 School of Science, Mae Fah Luang University, Chiang Rai, 57100 Thailand; 3 Center of Excellence in Fungal Research, Mae Fah Luang University, Chiang Rai, 57100 Thailand; 4 Institut Sophia Agrobiotech, UMR INRAE 1355, CNRS 7254, Université Côte d’Azur, Sophia Antipolis, Provence-Alpes-Côte d’Azur France

**Keywords:** Cross Kingdom RNA, Host induced gene silencing, Microbe induced gene silencing, Spray induced gene silencing, RNA Pesticides

## Abstract

Cross-kingdom RNA (CK-RNA) communication is an advanced concept that enhances our understanding of inter-kingdom interactions by uncovering RNA-mediated signaling among plants, fungi, bacteria, and viruses. This review provides a detailed overview of CK-RNA mechanisms, highlighting on plant–microbe interactions. Through RNA interference (RNAi), CK-RNA allows organisms to exchange regulatory signals that influence gene expression, defense responses, metabolism, and adaptive processes across kingdoms. Furthermore, this review highlights agricultural applications of CK-RNA, including Spray-Induced Gene Silencing (SIGS), Host-Induced Gene Silencing (HIGS) and Microbe-Induced Gene Silencing (MIGS) which offer environmentally friendly and sustainable alternatives to conventional chemical management. Overall, the integration of CK-RNAs as target and MIGS as delivery method presents a promising pathway for viable, environmentally friendly crop protection that aligns with modern goals of environmental sustainability and agricultural resilience.

**Graphical Abstract:**

**Figure:**
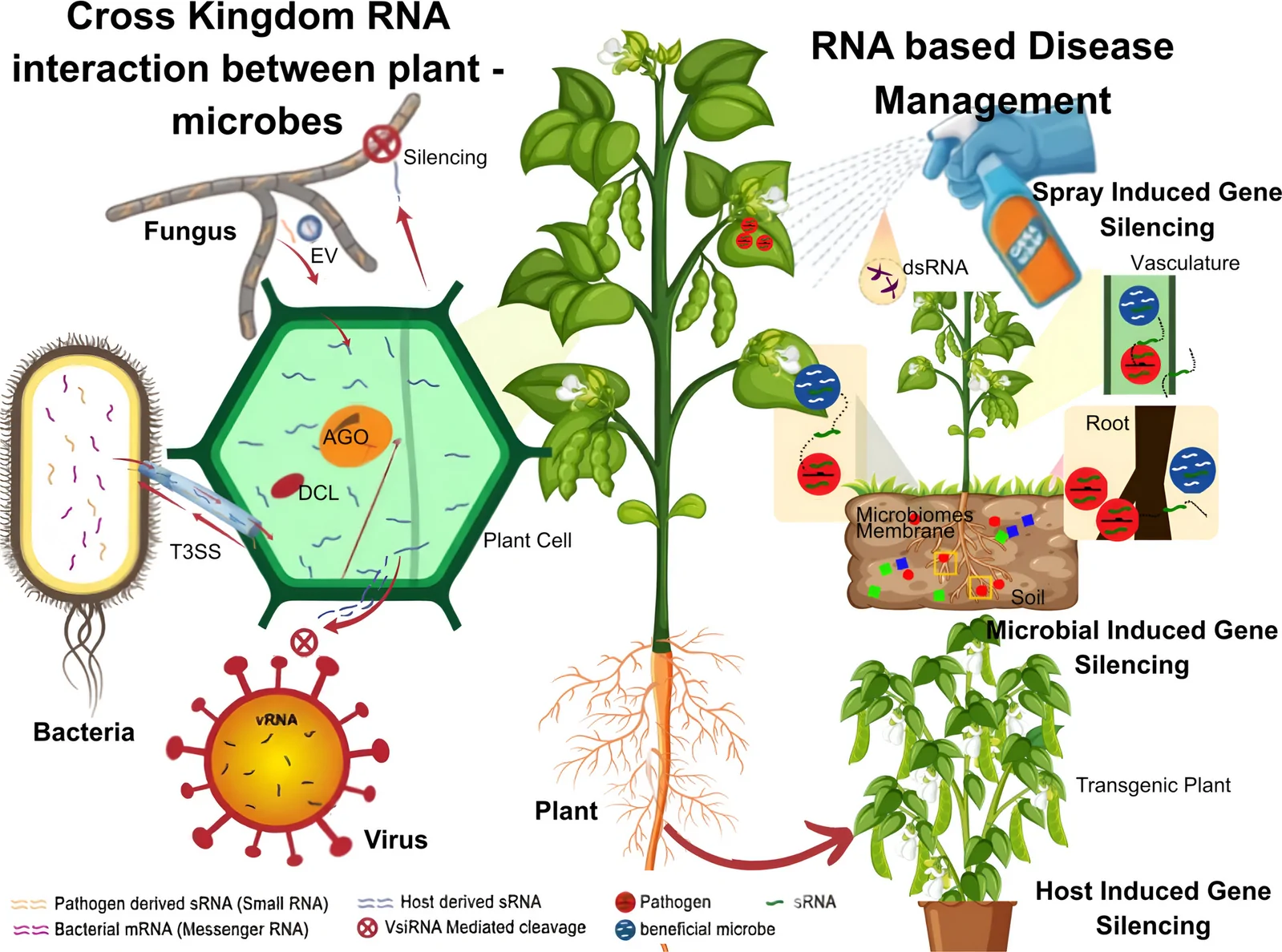

## Introduction

Ribonucleic acid (RNA) is a fundamental biomolecule involved in gene expression and regulatory processes across all domains of life (Brosius and Raabe 2016; Roundtree et al. 2017). In addition to its classical roles in transcription and translation, regulatory RNAs including small interfering RNAs (siRNAs), microRNAs (miRNAs), and long non-coding RNAs (lncRNAs) are key modulators of gene expression, epigenetic regulation, and cellular defense responses (Bhogireddy 2021, Yajnik et al. 2024), Beyond intracellular regulation, RNA molecules also participate in inter-organismal communication, forming the basis of cross-kingdom RNA (CK-RNA) signaling.

Building on these regulatory functions, RNA has been shown to function beyond the boundaries of a single organism through CK-RNA communication (Wang et al. 2024b; Piombo et al. 2024). CK-RNA communication refers to the transfer of small RNAs (sRNAs) including siRNAs and miRNAs, between different biological kingdoms to regulate gene expression and interspecies interactions (Cai et al. 2018b, a; Kapadia et al. 2025). In plant–microbe systems, CK-RNA exchange is increasingly recognized as a bidirectional regulatory mechanism. For instance, plants can deliver siRNAs to fungal pathogens, tosilencing virulence-related genes and reducing infection, while pathogens can transfer sRNAs into host plants to suppress immune responses and enhance colonization (Qiao et al. 2021; Cheng et al. 2023; Zhao et al. 2024). These RNA-mediated exchanges contribute to complex interspecies regulatory networks that shape ecological interaction and evolutionary dynamics across biological systems (Mahanty et al. 2023; Qin et al. 2023). These findings have led to the development of RNA-based agricultural strategies that exploit natural RNA-transfer mechanisms for crop protection.

Importantly, CK-RNA-mediated interactions are not restricted to pathogenic relationships but also occur in beneficial and symbiotic associations, indicating a broader role in plant-associated microbiomes. The ability of sRNAs to move across species boundaries highlights their potential as naturally evolved regulatory molecules that can be harnessed for agricultural applications. However, while CK-RNAs originate from long-term plant–microbe co-evolution and show sequence-specific regulatory activity, their ecological safety and functional stability under field conditions remain poorly understood.

The agricultural applications of CK-RNA have led to the development of RNA interference (RNAi) -based crop protection strategies, including host-induced gene silencing (HIGS), spray-induced gene silencing (SIGS), and microbe-induced gene silencing (MIGS), demonstrate its transformative potential (Zhao et al. 2024). These RNAi-based technologies enable sequence-specific targeting of pathogen genes, reducing reliance on conventional chemical control strategies (Mann et al. 2023). Although they share a common RNAi mechanism, these technologies differ in RNA origin, delivery mode, persistence, and field applicability.

Despite rapid progress in CK-RNA research and RNAi-based technologies, several key challenges remain unresolved. These include incomplete understanding of RNA transfer mechanisms across diverse plant–microbe systems, limited knowledge of RNA packaging, transport, and stability in extracellular environments, and insufficient comparative evaluation of SIGS, HIGS, and MIGS under realistic field conditions. In addition, mutualistic plant–microbe interactions remain underexplored compared with pathogenic systems, and the ecological relevance of CK-RNA-mediated communication across different biological contexts is still debated.

RNA-based biopesticides are rapidly emerging as a promising alternative to conventional crop protection strategies; however, their development is constrained by challenges in target gene selection, delivery optimization, and field-scale stability. In this context, this review synthesizes current knowledge on CK-RNA-mediated plant–microbe communication and critically evaluates SIGS, HIGS, and MIGS technologies, with the aim of providing a comprehensive framework for future research and the development of sustainable RNA-based agricultural applications.

## Cross-kingdom RNA in plant–microbe interactions

### CK-RNA in Plant-Fungal Interactions

#### Fungus-to-plant cross-kingdom RNA communication

Plant-fungal interactions are major targets of CK-RNA research due to the significant agricultural damage caused by fungal pathogens, many of which exhibit resistance to chemical fungicides (Mann et al. 2023; Schaefer et al. 2020). During infection, pathogenic fungi release sRNAs that enter host cells and specifically silence plant defense genes, thereby enhancing pathogen virulence (Hua et al. 2018). These fungal sRNAs act as CK-RNA effectors that modulate host immunity and physiology (Table 1).

**Table 1:** Cross-kingdom sRNAs in plant–microbe interactions

	**milRNAs/siRNA family**	**sRNA from**	**sRNA to**	**Disease**	**Cross-kingdom target**	**Reference**
Fungi to plants	Bc-sRNAs (Bc-siR3.1, Bc-siR3.2, Bc-siR5)	*Botrytis cinerea*	*Arabidopsis thaliana*, *Solanum lycopersicum* (Tomato)	Gray mold	Host immunity genes (via AGO1)	Weiberg et al. (2013)
	Pst-milR1	*Puccinia striiformis f.* sp.* tritici*	*Triticum aestivum (Wheat)*	Stripe rust	PR2 (pathogenesis-related 2)	Wang et al. (2017)
	Foc-milR87	*Fusarium oxysporum f.* sp.* cubense*	Musa acuminata (Banana)	Fusarium wilt	MaPTI6L (SA pathway regulator)	Zhong (2025)
	LtmilR2	*Lasiodiplodia theobromae*	*Vitis vinifera* (Grapevine)	Grapevine canker	LtRASGEF (fungal RAS signaling factor)	Huang et al. (2025)
	Vm-milR1	*Valsa mali*	*Malus domestica* (Apple)	Valsa canker	MdRLKT1, MdRLKT2 (receptor-like kinases)	Xu et al. (2022)
	Vm-milR1	*Valsa mali*	*Malus domestica* (Apple), *Pyrus* (Pear)	Valsa canker	MdRLKT1, MdRLKT2	Lei et al. (2025)
	Rhi-milR-13	*Rhizoctonia solani* AG1-1A	*Oryza sativa* (Rice)	Sheath blight	Vacuolar-sorting receptor precursor	Prathi et al. (2021)
	Rhi-milR-124	*Rhizoctonia solani* AG1-1A	*Oryza sativa* (Rice)	Sheath blight	KANADI1	Prathi et al. (2021)
	Rhi-milR-135	*Rhizoctonia solani* AG1-1A	*Oryza sativa* (Rice)	Sheath blight	Isoflavone reductase	Prathi et al. (2021)
	Rhi-milR-131	*Rhizoctonia solani* AG1-1A	*Oryza sativa* (Rice)	Sheath blight	Nuclear transcription factor Y	Prathi et al. (2021)
	Rhi-milR-18	*Rhizoctonia solani* AG1-1A	*Oryza sativa* (Rice)	Sheath blight	NB-ARC domain protein	Prathi et al. (2021)
	Rhi-milR-142	*Rhizoctonia solani* AG1-1A	*Oryza sativa* (Rice)	Sheath blight	F-box protein OsFBX438	Prathi et al. (2021)
	FsK-hpGF	*Fusarium solani strain K*	*Nicotiana benthamiana*	-	GFP reporter gene	Kellari et al. (2025)
	Rir2216	*Rhizophagus irregularis*	*Medicago truncatula*	Mutualistic Arbuscular mycorrhizal symbiosis	MtWRKY69 Transcription factor	Silvestri et al. (2025)
	Foc-milR138	*Fusarium oxysporum f.* sp.* cubense*	*Musa* spp. (Banana)	Fusarium wilt	MaLYK3 (receptor-like kinase)	He et al. (2024)
	Fol-milR1	*Fusarium oxysporum f.* sp.* lycopersici*	*Solanum lycopersicum* (Tomato)	Tomato wilt	SlyFRG4 (CBL-interacting protein kinase); interferes with SlyAGO4a	Ji et al. (2021)
Plant to fungi	Bd-sRNAs	*Brachypodium distachyon*	*Magnaporthe oryzae*	Rice blast	Cell wall genes: chitin deacetylase 1 (MGG_05023), cell wall protein (MGG_09460); Virulence genes: CAP20 (MGG_11916), CON7 (MGG_05287), AvrPiz-t (MGG_09055), SGA1 (MGG_01096), Sso1 (MGG_04090), YHM2 (MGG_07201), MoATG17 (MGG_07667)	Zanini et al. (2021)
	Hvu rRFs, milRNAs (Hvu rRF0001, Hvu rRF0002, Hvu rRF0003 + MYC/EPI/EV + -enriched milRNAs)	*Hordeum vulgare* (Barley)	*Blumeria hordei*	Powdery mildew	Microtubule severing, vacuole function, ATP/ADP binding, protein deubiquitination	Kusch et al. (2023)
	Barley miRNAs and phasiRNAs	*Hordeum vulgare* (Barley)	*Blumeria graminis f.* sp.* hordei*	Powdery mildew	EKA effectors (AVRk1/AVRa10), CSEP effector families	Hunt et al. (2019)
	SlmiRNA159, SlmiR162, SlsRNA4, SlsRNA11, SlsRNA12	*Solanum lycopersicum* (Tomato)	*Botrytis cinerea*	Gray Mold	Bcin12g05240, Bcin05g04730, Bcin07g06910, Bcin04g05820	Cheng (2025)
	Nb-miR172	*Nicotiana benthamiana*	*Fusarium verticillioides*	Fusarium infection	Fv-V-ATPase	Aydinoglu and Kuloglu (2023)
	miR166, miR159	*Gossypium hirsutum* (Cotton)	*Verticillium dahliae*	Vascular wilt	Clp-1 (miR166), HiC-15 (miR159)	Zhang et al. (2016)
Plant to bacteria	Plant sRNAs (TRV-mediated)	*Ralstonia pseudosolanacearum*	*Nicotiana benthamiana*	Bacterial wilt	Virulence genes & GFP	Jang et al. (2024)
	IR-CFA6/HRPL	*Pseudomonas syringae*	*Arabidopsis thaliana*	Reduced pathogenesis	cfa6, hrpL	Ravet et al. (2025)
Bacteria to plants	Xosr001	*Xanthomonas oryzae pv. oryzicola*	*Oryza sativa* (rice)	Bacterial leaf streak	OsJMT1 (affecting MeJA and stomatal immunity)	Wu et al. (2024)

*Botrytis cinerea* secretes sRNAs such as Bc-siR3.1, Bc-siR3.2, and Bc-siR5 into plant cells, where they hijack the host AGO1 complex to silence immunity-related genes (Weiberg et al. 2013). Similarly, the rust fungus *Puccinia striiformis* delivers milRNA Pst-milR1 into wheat to suppress the defense gene PR2, which encodes the β−1,3-glucanase involved in fungal cell wall degradation (Wang et al. 2017). In *F. oxysporum f.* sp.* cubense*-banana system, Foc-milR87 targets the transcriptional activator MaPTI6L, reducing expression of the salicylic acid marker MaEDS1 and impairing early defense responses (Zhong 2025). Another fungal sRNA, Foc-milR138, targets MaLYK3, the receptor-like kinase in banana and disrupts immune signaling (He et al. 2024). Likewise, Fol-milR1 from *F. oxysporum f.* sp.* lycopersici* suppresses tomato wilt resistance by silencing the CBL interacting protein kinase, SlyFRG4, and interfering with the host Argonaute 4a, SlyAGO4a (Ji et al. 2021). Collectively, these studies demonstrate a conserved mechanism by which *Fusarium* species translocate milRNAs into host cells to manipulate defense-related genes. This highlights the vital role of CK-RNA mediated for RNAi in fungal pathogenicity.

CK-RNA regulation is also observed in rice–*Rhizoctonia solani* interactions, where Rhi-milR-13, Rhi-milR-135, Rhi-milR-131, Rhi-milR-18, and Rhi-milR-142 silence multiple rice defense regulators. These include vacuolar sorting receptors, isoflavone reductase, NF-Y transcription factors, NB-ARC/NLR immune receptors, and F-box proteins. This results in the suppression of hypersensitive response and multiple layers of immunity (Prathi et al. 2021). In apple, *Valsa mali* uses the milRNA Vm-milR1 to silence receptor-like kinase genes *MdRLKT1* and *MdRLKT2*, reducing ROS accumulation, callose deposition, and defense gene expression (Xu et al. 2022). In a subsequent study, the apple susceptibility-related receptor MdRLKT21 was shown to bind with Vm-milR1, relieving the suppression of MdRLKT1 and MdRLKT2, and activating the C3HC4-type RING finger protein MdRFP1, thereby enhancing resistance (Lei et al. 2025). These findings highlight the dynamic and reciprocal regulation between fungal sRNAs and plant receptors.

CK-RNA signaling also operates in beneficial interactions. In *Medicago truncatula*, the arbuscular mycorrhizal (AM) fungus *Rhizophagus irregularis* delivers sRNA Rir2216, which hijacks AGO1 to repress MtWRKY69, thereby promoting mutualistic colonization (Silvestri et al. 2025). Additionally, *Fusarium solani* strain K exports sRNAs into *Nicotiana benthamiana*, triggering systemic RNA silencing via RDR6-dependent secondary siRNA amplification (Kellari et al. 2025). Together, these findings demonstrate that CK-RNA trafficking is a conserved communication strategy in both pathogenic and mutualistic fungi.

#### Cross-kingdom RNA from plant-to-fungus

Bidirectional RNA trafficking is now recognized as a central mechanism in plant–fungus interactions. In addition to fungal sRNAs entering plant cells, plants also export their own sRNAs into fungal pathogens, whereby they silence genes essential for virulence (Table 1).

These plant-derived sRNAs are delivered via extracellular vesicles (EVs) or direct cellular interfaces (e.g., haustoria). Plants selectively package sRNAs into EVs through the action of RNA-binding proteins, including AGO1, RH11, RH37, ANN1, and ANN2, which contribute to RNA sorting, stabilization, and transport (He et al. 2021). Encapsulation within EVs protects RNA cargo from extracellular nucleases and other environmental factors that could otherwise compromise RNA stability during interspecies transfer (Zhao et al. 2024; Li et al. 2024). Recent evidence indicates that fungal EVs can deliver molecular cargo into plant cells through clathrin-mediated endocytosis, highlighting the importance of membrane trafficking pathways in CK-RNA communication (Wang et al. 2025a, b). In addition to receiving plant-derived RNAs, fungi can secrete their own EV-associated sRNAs that are delivered into host cells to suppress plant immunity and facilitate infection (He et al. 2021; Cai 2021). These findings highlight EVs as important vehicles for intercellular and cross-kingdom molecular communication, while several mechanistic aspects of EV biology remain poorly understood, including cargo loading, recipient recognition, uptake pathways, and transport efficiency (Zaheer 2025).

In *Brachypodium distachyon*–*Fusarium graminearum* system, 258 plant-derived CK-RNA sRNAs were identified. These sRNAs target fungal mRNAs and significantly reduce pathogenicity (Werner et al. 2025). These findings highlight the potential of endogenous plant sRNAs as highly specific and effective bioprotectants against fungal diseases. In another example, wheat roots produce specific miRNAs that enter the biocontrol fungus *Clonostachys rosea* and downregulate genes such as pks29, which modulate fungal secondary metabolism. Rather than activating defense, these plant-derived sRNAs fine-tune the physiology of beneficial fungi (Piombo et al. 2024), highlighting the broader roles of sRNAs in shaping plant microbe interactions beyond pathogenicity.

Understanding the bidirectional movement of CK-RNA in plant-to-fungus and fungus-to-plant communication forms a foundation for studying similar mechanisms in other plant–microbe systems. CK-RNA communication is not limited to fungi; bacteria and viruses also use RNA-based pathways to influence plant immunity, growth, and colonization. Elucidating these mechanisms is critical for developing sustainable agricultural strategies to enhance crop resilience in the face of climate change and rising pathogen pressures.

### Plant–bacteria cross-kingdom RNA communication

Plant–bacteria interactions represent RNA-regulated communication systems within plant-associated microbiomes. Beyond classical chemical signaling, sRNAs and miRNAs have emerged as regulatory molecules capable of mediating inter-organismal communication. Evidence suggests that bacterial sRNAs and plant-derived regulatory RNAs can reciprocally influence gene expression, adding an additional regulatory layer to plant–microbe interactions (Zhang et al. 2024; Palermo and Weiberg 2025).

Taken together, current evidence indicates that RNA-mediated communication occurs in both pathogenic and beneficial plant–bacterial interactions. However, mechanistic evidence remains considerably stronger for pathogenic systems than for mutualistic associations. Future studies should focus on understanding how RNAs are selectively packaged, transported, and recognized by recipient cells, as these processes remain major knowledge gaps in the field.

#### Bacteria-to-plant cross-kingdom interactions

Bacterial pathogens can deploy sRNAs as inter-organismal effectors that reprogram plant immune responses. A major delivery route involves outer membrane vesicles (OMVs), which protect RNA cargo and facilitate transfer into plant tissues (Yu et al. 2022).

In *Xanthomonas oryzae* pv. *oryzicola*, the OMV-associated sRNA Xosr001 is delivered into rice cells, where it targets *OsJMT1*, a jasmonate metabolism gene. This interaction alters jasmonic acid signaling and suppresses stomatal immunity (Wu et al. 2024). This example demonstrates that bacterial sRNAs can function as mobile regulatory effectors capable of modulating plant hormonal and immune pathways following transfer into host tissues.

Beyond RNA cargo, OMVs are increasingly recognized as multifunctional vehicles that contribute to host immune modulation and inter-organismal signaling (McMillan et al. 2021). These findings suggest that bacterial sRNAs can function as mobile regulatory molecules that influence plant defense networks, although mechanistic understanding remains largely restricted to pathogenic systems.

#### Plant-to-bacteria RNA communication

Plants release extracellular RNAs into the apoplast, the primary interface for plant–bacteria interactions, enabling RNA-based inter-organismal communication (Kaffarnik et al. 2009; Borniego et al. 2025). These RNAs are transported via extracellular vesicle-dependent and vesicle-independent pathways and can be taken up by bacterial cells, where they may regulate gene expression in a sequence-specific manner (Palermo and Weiberg 2025).

Functional evidence for this mechanism has been demonstrated in *Arabidopsis thaliana*, where HIGS targeting *Pseudomonas syringae* virulence genes (*Cfa6* and *HrpL*) reduced bacterial colonization and impaired stomatal reopening. These antibacterial RNAs were delivered via both TET8-associated Evs and PEN1-associated extracellular protein fractions, indicating multiple RNA export routes (Ravet et al. 2025).

Similarly, plant-derived sRNAs can suppress gene expression in *Ralstonia pseudosolanacearum*, indicating that RNA-mediated regulation extends across diverse bacterial species (Jang et al. 2024). Collectively, these findings support a model in which plant-derived sRNAs function as mobile regulatory molecules that directly target bacterial gene expression as part of an RNA-based defense strategy.

#### RNA communication in beneficial plant–bacterial associations

RNA-mediated regulatory interactions are increasingly recognized in beneficial plant–bacterial associations, although mechanistic evidence for bidirectional RNA transfer remains limited.

In rhizobium–legume symbiosis, plant miRNAs regulate nodulation and nitrogen fixation processes through conserved pathways such as miR160–ARF, miR171–NSP2, and miR172–AP2 (Hoang et al. 2020; Tiwari et al. 2021). Importantly, *Bradyrhizobium japonicum* produces tRNA-derived fragments (tRFs) that accumulate in soybean nodules and utilize the host AGO1 machinery to regulate plant transcripts involved in nodulation, providing direct evidence of bacterial RNA influence in mutualistic symbiosis (Ren et al. 2019).

Plant growth-promoting rhizobacteria (PGPR) can also modulate host small RNA networks associated with abiotic stress responses. In chickpea, inoculation with *Pseudomonas putida* RA alters the expression of conserved stress-responsive miRNAs, including miR159, miR160, miR166, miR167, miR169, miR171, and miR396, along with corresponding target gene regulation under drought and salinity stress (Yadav et al. 2023). However, whether these changes result from direct RNA transfer or indirect hormonal signaling remains unclear. Compared with pathogenic interactions, evidence for naturally occurring RNA exchange in beneficial plant–bacterial associations remain limited, highlighting an important area for future research.

More recently, beneficial bacteria have been shown to act as RNA delivery vehicles. Engineered strains of *Bacillus subtilis* and *Pseudomonas putida* can package dsRNAs into EVs and deliver them to fungal pathogens, suppressing infection (Niño-Sánchez et al. 2026). Collectively, these findings suggest that beneficial microorganisms can influence host gene regulation through RNA-based mechanisms, although natural RNA trafficking between PGPR and plants requires further validation.

Despite strong evidence for RNA-mediated regulation in plant–virus systems, whether these interactions fully represent CK-RNA transfer remains debated. Unlike plant–fungal or plant–bacterial systems, viral RNA is continuously replicated within host cells, making it difficult to distinguish between inter-organism RNA transfer and intracellular RNA processing events. Moreover, most current studies are based on controlled laboratory infections, and the ecological relevance of virus-derived small RNA movement under field conditions is still unclear. Therefore, plant–virus systems are best interpreted as a closely related but mechanistically distinct model of RNA-based host–pathogen regulation.

### RNA-mediated communication in plant–virus interactions

Viruses are not classified within the traditional biological kingdoms, however plant–virus interactions provide a well-characterized system for studying RNA-mediated host–pathogen regulation (Bimrew and Abera 2023). Viruses extensively manipulate host gene expression, while plants rely on RNA silencing pathways as a primary antiviral defense mechanism (Deng et al. 2022; Kutnjak et al. 2015). Therefore, plant–virus systems are included in this review as functionally relevant models of RNA-mediated regulatory interactions that resemble CK-RNA dynamics.

#### Plant-to-virus RNA communication: host antiviral defense

RNAi is a key antiviral defense mechanism used by plants. Viral double-stranded RNA is processed by Dicer-like (DCL) enzymes into virus-derived small interfering RNAs (vsiRNAs), which are loaded into AGO proteins to form RNA-induced silencing complexes (RISCs). These complexes guide sequence-specific degradation of viral RNAs, limiting viral replication and spread (Ramesh 2021; Pang 2022; Koloniuk et al. 2023).

This RNAi response is dynamic and involves extensive remodeling of host small RNA populations during infection. The production of diverse vsiRNAs enables simultaneous targeting of multiple viral genomic regions, providing a robust and adaptable antiviral defense system (Kutnjak et al. 2015).

#### Virus-to-plant RNA communication: viral manipulation of host gene expression

Viruses can suppress and reprogram host RNA silencing pathways, partly by changing host miRNA levels. For example, Rice stripe virus (RSV) infection increases some miRNAs such as miR444 and miR168, while reducing others involved in plant defense and development, thereby affecting host gene regulation (Yang et al. 2016).

Viruses also encode RNA silencing suppressor proteins that interfere with host RNAi machinery. The RSV-encoded protein NS3 binds sRNAs to suppress antiviral silencing and interacts with DRB1 to modulate miRNA biogenesis, thereby simultaneously inhibiting defense pathways and altering host regulatory circuits (Zheng et al. 2017).

In addition, virus-derived sRNAs can directly regulate host transcripts. Rice black-streaked dwarf virus (RBSDV) produces vsiRNAs that target host genes in rice and maize, demonstrating direct viral RNA-mediated regulation of plant gene expression (Wang et al. 2024a). Furthermore, virally activated siRNAs (vasiRNAs), generated through DCL4-, RDR1-, and AGO2-dependent pathways, extend RNA-mediated regulation to endogenous plant transcripts during infection (Zhang et al. 2019).

Virus infection can also extend beyond plant hosts. For example, Cucumber mosaic virus (CMV) has been reported to infect the phytopathogenic fungus *Rhizoctonia solani*, suggesting that virus-associated RNA interactions may occur in broader multi-organismal networks (Andika 2017).

#### Bidirectionality and complexity of plant–virus RNA interactions

Plant–virus interactions represent a dynamic system of bidirectional RNA-mediated regulation between host and virus (Lopez-Gomollon and Baulcombe 2022). Plants use vsiRNA-guided silencing pathways to target viral RNAs, while viruses respond by suppressing these defenses and producing their own regulatory RNAs that alter host gene expression (Zhu and Guo 2012).

Genome-wide analyses of Turnip mosaic virus (TuMV)-infected *Brassica napus* reveal complex RNA regulatory networks involving both virus-derived vsiRNAs and host-derived vasiRNAs. Viral siRNAs can target host transcripts, while host-derived vasiRNAs regulate both viral and endogenous plant genes, illustrating multilayered RNA-guided control during infection (Pitzalis et al. 2020).

In addition to RNAi, epitranscriptomics regulation contributes to antiviral defense. For example, m6A modification of Potato virus Y RNA by the host methyltransferase NbMTA promotes viral RNA degradation and restricts infection (Li et al. 2025). Although virus-associated RNA processes do not strictly meet the classical definition of CK-RNA transfer, they represent a functionally analogous system in which RNA molecules mediate bidirectional regulatory interactions between host and pathogen. Therefore, plant–virus systems are included in this review as representative models of RNA-mediated host–pathogen communication.

## Applications of cross-kingdom RNA in agriculture

CK-RNA communication is a powerful biological principle with direct applications in modern agriculture (Rabuma et al. 2022; Zeng et al. 2019). As plants increasingly face pressure from climate change, emerging pathogens, and the need for reduced chemical inputs, RNA-based strategies provide a precise and sustainable alternative to conventional crop protection (Zhao et al. 2024). CK-RNA mechanisms, whether naturally occurring or synthetically engineered (Zeng et al. 2019), enable targeted regulation of gene expression across species boundaries, offering new ways to suppress pathogens, modulate plant immunity, and shape host-microbe interactions. These insights have demonstrated the development of innovative technologies such as SIGS, HIGS, and MIGS. These three approaches represent different delivery strategies of a shared RNAi mechanism, differing primarily in RNA origin, stability, and field applicability.

This section outlines the major advances in agricultural applications of CK-RNA and highlights how these approaches can transform pathogen and disease management into sustainable agriculture.

### Spray induced gene silencing

Spray Induced Gene Silencing (SIGS) is a novel, non-transgenic RNAi approach in which plant surfaces are sprayed with dsRNAs or siRNAs designed to silence essential genes in pests and pathogens (Wang and Jin 2018, Sarkar and Roy-Barman 2021). After application, dsRNAs can be absorbed by plant tissues, and subsequently suppress invading pathogens (Mitter 2017; Mosquera et al. 2025).

SIGS is highly versatile, functioning across different biological kingdoms (Zeng et al. 2019). SIGS has been widely evaluated for controlling fungal diseases, primarily by targeting virulence-associated genes (Table 2). Topical applications of dsRNAs have been shown to effectively suppress diseases such as gray mold, stem rot, and wilt (Qiao et al. 2021; Spada et al. 2025). The efficiency of SIGS is strongly influenced by the ability of pathogens to uptake the RNA, which directly affects gene silencing efficacy (Qiao et al. 2021).

**Table 2:** RNA based disease management strategies (SIGS and HIGS)

siRNA/dsRNA family	sRNA from	sRNA to	Disease	Cross-kingdom target	References
dsRNA targeting VPS51, DCTN1, SAC1, DCL1/2 genes	Lettuce, Tomato, Grape, Rose	*Botrytis cinerea*	Gray mold	VPS51, DCTN1, SAC1, DCL1/2	Qiao et al. (2021)
dsRNA targeting VPS51, DCTN1, SAC1, DCL1/2 genes	Lettuce, Collard Greens	*Sclerotinia sclerotiorum*	White mold	VPS51, DCTN1, SAC1, DCL1/2	Qiao et al. (2021)
dsRNA targeting VPS51, DCTN1, SAC1, pgxB genes	Tomato, Apple, Grape	*Aspergillus niger*	Fruit rot/Black mold	VPS51, DCTN1, SAC1, pgxB	Qiao et al. (2021)
dsRNA targeting DCTN1, SAC1, PG genes	Rice	*Rhizoctonia solani*	Sheath blight	DCTN1, SAC1, PG	Qiao et al. (2021)
dsRNA targeting DCL1/2, DCTN1, SAC1 genes	Arabidopsis	*Verticillium dahliae*	Verticillium wilt	DCL1/2, DCTN1, SAC1	Qiao et al. (2021)
dsRNA targeting VPS51, DCTN1, SAC1, DCL1/2 genes	Tomato	*Botrytis cinerea*	Gray mold (SIGS longevity test)	VPS51, DCTN1, SAC1, DCL1/2	Qiao et al. (2021)
dsRNA targeting BcBmp1, BcBmp3, BcPls1 genes	Lettuce (*Lactuca sativa*)	*Botrytis cinerea*	Gray mold disease	BcBmp1, BcBmp3, BcPls1	Spada et al. (2025)
siRNA and dsRNA targeting CYP51; dsRNA targeting AATF, LIP1, LIPA, ACX, NCED, ABA2, ABAR, and EC2 genes	*Arabidopsis thaliana*	*Golovinomyces orontii*	Powdery mildew	CYP51, AATF, LIP1, LIPA, ACX, NCED, ABA2, ABAR, EC2	McRae (2023)
dsRNA targeting *CYP51*, *AATF*, *LIP1*, *LIPA*, *NCED*, and *EC2 genes*	*Vitis vinifera* (grapevine)	*Erysiphe necator*	Powdery mildew	CYP51, AATF, LIP1, LIPA, NCED, EC2	McRae (2023)
dsRNAtargeting *β-tubulin*, *CYP51*, and *Chs* genes	*Hevea brasiliensis* (Rubber tree)	*Erysiphe quercicola*	Powdery mildew	β-tubulin, CYP51 (two fragments), Chitin synthase (Chs)	Cao et al. (2024)
dsRNA targeting *PiGPB1*, *PiHmp1*, *PiCut3*, and *PiEndo3* genes	Potato/Tomato (Solanum tuberosum)	*Phytophthora infestans*	Late blight	PiGPB1, PiHmp1, PiCut3, PiEndo3	Kalyandurg et al. (2021)
dsRNA targeting *Fg10360*, *Fg13150*, and *Fg06123 genes*	Barley	*Fusarium graminearum*	Fusarium head blight/leaf infection	Fg10360, Fg13150, Fg06123	Kim et al. (2023)
dsRNA targeting *Vps51*, *DCTN1*, *SAC1*, *Pp2a*, *Sit4*, *Ppg1*, *Tap42*, *Chs1*, *Chs2*, *Chs3b*, and *Gls1 genes*	Pine tree	*Fusarium circinatum*	Pine Pitch Canker Disease	Vps51, DCTN1, SAC1, Pp2a, Sit4, Ppg1, Tap42, Chs1, Chs2, Chs3b, Gls1	Bocos-Asenjo (2025)
dsRNA targeting *RcOSP1 genes*	Wheat (Wenmai 6)	*Rhizoctonia cerealis*	Sharp eyespot	RcOSP1	Li et al. (2022)
dsRNA targeting *SsOah1* (SS1G_08218) and *SsCyp51* (SS1G_04805) genes	*Brassica juncea*	*Sclerotinia sclerotiorum*	Sclerotinia Stem Rot (SSR)	SsOah1 (SS1G_08218) & SsCyp51 (SS1G_04805)	Pant and Kaur (2023)
dsRNA targeting *RiRgs3*, *RiGpa3*, and *RiGpb1* (G-protein signaling genes) genes	Various land plants (AM symbiosis)	*Rhizophagus irregularis*	AM symbiosis	RiRgs3, RiGpa3, RiGpb1	Fan et al. (2025)
In vivo-synthesized dsRNA targeting *UvCYP51*, *UvBI-1*, and *UvbZIP11 genes*	*Oryza sativa* (rice)	*Ustilaginoidea virens*	Rice False Smut (RFS)	UvCYP51, UvBI-1, UvbZIP11	Zhang et al. (2024)
dsRNA targeting *SsAgo2* gene	*Nicotiana benthamiana*/Sunflower	*Sclerotinia sclerotiorum*	Sclerotinia rot	SsAgo2 (PIWI/RNaseH domain)	Mukherjee et al. (2024)
dsRNA-FsCYP51A/B/C	*Fusarium sacchari*	Sugarcane	Pokkah boeng disease	FsCYP51A, FsCYP51B, FsCYP51C	Yin et al. (2025)
RNAi-FfCNA1/CNB1	*Fusarium fujikuroi*	Rice	Bakanae disease	FfCNA1, FfCNB1	Hou et al. (2025)
RNAi-BdSTP/ALS	*Botryosphaeria dothidea*	*Malus hupehensis*	Apple ring rot	BdSTP, BdALS (acetolactate synthase)	Yu et al. (2024)
RNAi-ALS	*Phytophthora infestans*	Potato	Late blight	ALS (acetolactate synthase)	Temme et al. (2025)
RNAi-NLP1	*Verticillium dahliae*	Arabidopsis	Verticillium wilt	NLP1 (necrosis- and ethylene-inducing protein-like 1)	Song and Thomma (2018)
RNAi-Sge1	*Verticillium dahliae*	Arabidopsis	Verticillium wilt	Sge1 (transcription factor for virulence)	Song and Thomma (2018)
RNAi-FcGls1	*Fusarium culmorum*	Wheat	Fusarium head blight	FcGls1 (β−1,3-glucan synthase)	Chen (2016)
RNAi-FcGls1 +	*Fusarium culmorum*	Wheat	Fusarium head blight	FcGls1 + virulence genes	Chen (2016)
RNAi-SsGlcP	*Sporisorium scitamineum*	Sugarcane	Smut disease	SsGlcP (β−1,6-glucanase precursor)	Wu et al. (2025)
dsRNA-RGS1/APT2/LHS1	*Magnaporthe oryzae*	Rice	Blast disease	RGS1, MgAPT2, LHS1 pathogenicity genes	Jin et al. (2024)
RNAi-SsMPG1/MPG2	*Sclerotinia sclerotiorum*	*Nicotiana benthamiana*, *Arabidopsis* *thaliana*	Sclerotinia stem rot	SsMPG1, SsMPG2	Zhang et al. (2025)
RNAi-MoMTG1	*Magnaporthe oryzae*	Rice	Blast disease	MoMTG1	Wang et al. (2025c)
dsRNA—NOB1	*Sclerotinia sclerotiorum*	*Arabidopsis thaliana*	Sclerotinia stem rot	NOB1 (SS1G_07873) + interacting genes	Caners (2025)
Bc-sRNAs	*Botrytis cinerea*	*Arabidopsis thaliana* (transgenic)	Gray mold/Downy mildew	STTM plants block these sRNAs, reducing infection	Cheng et al. (2023)

In wheat, topical application of dsRNAs delivered either alone or combined with nanocarriers reduced Fusarium head blight (FHB) severity, supporting SIGS as a sustainable alternative to conventional fungicides (Imran et al. 2025; Sundararajan et al. 2025). Similarly, dsRNA-mediated suppression of the RcOSP1 effector reduced sharp eyespot severity by limiting fungal virulence (Li et al. 2022). Encapsulation technologies, such as artificial vesicles (AVs), enhance dsRNA stability, protect against environmental degradation, and extend efficacy (Imran et al. 2025). For example, AV encapsulated dsRNAs targeting *B. cinerea* virulence genes provided extended protection for up to 10 days in tomato and grapes fruits and grape leaves up to 21 days in both pre- and post-harvest tissues (Qiao et al. 2023).

SIGS also served as a tool for dissecting host–pathogen interactions (Pant and Kaur 2023; Zhang et al. 2024). In *Brassica juncea* and *N. benthamiana,* silencing of *SsOah1* and *SsCyp51* delayed Sclerotinia stem rot progression and altered fungal morphology (Pant and Kaur 2023; Zhang et al. 2024). In lettuce, dsRNAs targeting *BcBmp1*, *BcBmp3*, and *BcPls1* significantly reduced gray mold severity, with nanosheet-based delivery further enhancing dsRNA stability and protection (Spada et al. 2025). Powdery mildew control in *Arabidopsis* and grapevine was achieved by targeting *CYP51*, *AATF*, lipid metabolism genes, and fungal effectors, demonstrating the versatility of SIGS for silencing multiple genes across phylogenetically distinct pathogens (McRae 2023). Similarly, SIGS reduced powdery mildew severity in rubber trees, suppressed pathogen genes in potato, and effectively suppressed pathogen development and reduced late blight severity by producing exogenous dsRNAs targeting *P. infestans* genes *PiGPB1, PiHmp1, PiCut3*, and *PiEndo3* (Kalyandurg et al. 2021). Repeated dsRNA applications targeting genes associated with vesicle trafficking (*Vps51, DCTN1, SAC1*), signaling pathways *(Pp2a, Sit4, Ppg1, Tap42*), and cell wall biosynthesis (*Chs1, Chs2, Chs3b, Gls1*) also mitigated pine pitch canker disease (Bocos-Asenjo 2025).

SIGS has also been applied to study beneficial plant microbe interactions, providing insight into symbiotic development and functional genomics (Vetukuri et al. 2025). Targeting AM fungal genes *RiRgs3*, *RiGpa3*, and *RiGpb1* impaired spore germination and hyphopodium formation, demonstrating G-protein signaling as essential for early symbiotic development (Fang et al. 2025; Fan et al. 2025). Additionally, SIGS has been used to suppress rice false smut by targeting *UvCYP51, UvBI-1*, and *UvbZIP11* (Zhang et al. 2024) and to control white mold in sunflower and *N. benthamiana* by silencing *SsAgo2,* combined with layered double-hydroxide nanosheet carriers further enhancing stability and delivery (Mukherjee et al. 2024).

Table 2 provides a summary of selected applications of SIGS in various crop–pathogen model systems, documenting targeted genes, host crops, pathogens, and observed biological outcomes. Analysis of these studies reveals consistent trends regarding the practical use of SIGS in crop protection.

*Graminearum*, *Sclerotinia sclerotiorum*, and *P. infestan* across diverse crops such as cereals (wheat, barley), vegetables (tomato, lettuce, potato), fruits (grape), and oilseeds (Brassica spp.) and ornamentals. Targeted genes include virulence factors, essential metabolic enzymes (e.g., CYP51 family), signaling regulators, and pathogen-specific effectors, emphasizing the precision and specificity of RNAi-based silencing. Delivery methods such as foliar spraying, encapsulation in nanocarriers, or artificial vesicles improve dsRNA stability, uptake efficiency, and prolong the gene-silencing effects.

SIGS offers several advantages over traditional chemical management. It is eco-friendly, non-transgenic, highly gene specific, and compatible with Integrated Pest Management (IPM) (Chen et al. 2025b; Beernink 2024; Mosquera et al. 2025). This method is highly adaptable, effective against a wide range of organisms, and compatible with sustainable agricultural practices (Chen et al. 2025b; Xi et al. 2025).

Despite its advantages, SIGS faces challenges due to the instability of naked RNA molecules, which rapidly degrade under UV light, nuclease activity, and environmental stresses (Beernink 2024; Qiao et al. 2024). Continued advances in protective delivery systems such as nanosheets, liposomes, or polymers can improve RNA stability and uptake. Future research should focus on optimizing delivery methods, reducing RNA production costs and understanding RNA uptake and transport across diverse pathogens (Vetukuri et al. 2025; Chen et al. 2025b).

While SIGS provides rapid and flexible deployment, its transient nature has led to the development of more durable in planta approaches such as HIGS.

### Host-Induced Gene Silencing (HIGS)

While SIGS relies on external application of RNA molecules, HIGS provides an internal, plant-encoded RNAi mechanism offering more durable protection (Zand Karimi and Innes 2022). HIGS is a CK-RNA, RNAi mechanism in which plants produce double-stranded RNAs (dsRNAs) that are processed into siRNAs capable of moving into invading pathogens. These siRNAs silence target genes and suppress infection (Mei et al. 2024). HIGS functions both as a functional genomics tool and a promising sustainable crop protection strategy (Sang and Kim 2020).

Transient HIGS approaches have been widely used to explore gene function and plant-pathogen interactions (Beernink 2024; Rajam and Chauhan 2021). Silencing the high-affinity nicotinic acid transporter gene *VdNAT1* in *Verticillium dahliae* inhibited fungal biomass accumulation and alleviated Verticillium wilt symptoms in cotton. This study demonstrates the potential of transient HIGS for functional studies and disease control (Wang et al. 2025a). HIGS has also been used to study beneficial fungi silencing the sucrose transporter gene *GspSUT1* in *Gongronella butleri* altered carbon allocation in *Actinidia* roots and affected soil nitrogen-fixing bacteria, demonstrating HIGS influence on plant growth and soil microbiome composition, beyond pathogen control (Fang et al. 2025). In root diseases transient HIGS targeting *Plasmodiophora brassicae* effectors Pb48 and Pb52 in *Brassica rapa* reduced root gall formation and modulated hormone-associated signaling pathways, demonstrating its utility of in susceptible hosts lacking stable transgenic lines (Yang et al. 2025).

Stable transgenic HIGS applications have demonstrated long-term resistance to major crops (Table 2). In sugarcane, transgenic expression of dsRNAs targeting the ergosterol biosynthesis genes *FsCYP51A, FsCYP51B*, and *FsCYP51C* significantly reduced the severity of Pokkah boeng disease (Yin et al. 2025) and RNAi-mediated silencing of *SsGlcP* impaired *Sporisorium scitamineum* infection, mitigating smut disease (Wu et al. 2025). Similarly, rice HIGS lines expressing RNAi constructs targeting *FfCNA1* and *FfCNB1* showed enhanced resistance to Bakanae disease caused by *Fusarium fujikuroi* (Hou et al. 2025). In apple, dsRNA constructs targeting *BdSTP* and *BdALS* suppressed *Botryosphaeria dothidea* infection and alleviated ring rot symptoms (Yu et al. 2024). Similarly, potato expressing hairpin RNAs targeting *ALS* gene exhibited durable resistance against *Phytophthora infestans* and late blight (Temme et al. 2025). *Arabidopsis* HIGS lines targeting *V. dahliae* virulence genes *NLP1* and *Sge1* effectively reduced Verticillium wilt progression (Song and Thomma 2018). In Cereals, wheat plants expressing RNAi constructs against *FcGls1*, alone or in combination with other virulence genes, displayed reduced Fusarium head blight symptoms caused by *Fusarium culmorum* (Chen 2016). Rice HIGS lines targeting pathogenicity genes *RGS1, MgAPT2,* and *LHS1* conferred resistance against rice blast disease (*Magnaporthe oryzae)* (Jin et al. 2024; Wang and Dean 2022). Similarly, *N. benthamiana* and *Arabidopsis* expressing hairpin RNAs against *SsMPG1* and *SsMPG2* demonstrated reduced Sclerotinia stem rot severity (Zhang et al. 2025), while *Arabidopsis* lines targeting *NOB1* and its interacting genes also showed suppression of disease progression (Caners 2025). Transgenic Arabidopsis plants expressing short tandem target mimics (STTM) effectively blocked *B. cinerea* sRNAs (Bc-sRNAs) that suppress host immunity, reducing infection by gray mold and downy mildew (Cheng et al. 2023).

HIGS provides a robust, gene-specific, and durable protection by enabling plants to continuously produce siRNAs targeting essential fungal genes, reducing reliance on chemical fungicides and can silence core metabolic genes (e.g., *ALS* in *P. infestans*) or virulence-associated genes (e.g., effector genes in *M. oryzae*), and support broad-spectrum or quantitative resistance (Govindarajulu et al. 2015; Temme et al. 2025).

However, HIGS faces several challenges. RNA uptake efficiency varies widely among pathogens, resulting in inconsistent silencing outcomes (Zand Karimi and Innes 2022, Temme et al. 2025). Transgenic crops face biosafety, regulatory restrictions, and public concerns and off-target effects may occur if target sequences overlap with genes from non-target organisms (Govindarajulu et al. 2015). Additionally, the development of stable transgenic lines requires substantial time and resources, and pathogen populations may evolve resistance, when single genes are targeted (Nowara et al. 2010).

Despite sharing a conserved RNAi mechanism, SIGS, HIGS, and MIGS differ fundamentally in their mode of RNA delivery, durability, and translational feasibility. SIGS depends on external application of dsRNA molecules, which allows rapid deployment and flexibility but is limited by environmental instability and variable uptake efficiency in target pathogens. In contrast, HIGS relies on endogenous expression of RNAi constructs within transgenic plants, enabling continuous and systemic gene silencing with higher durability, although its application is constrained by regulatory restrictions, biosafety considerations, and the time required for stable transformation. MIGS represents a more recent strategy that utilizes beneficial microorganisms as biological vectors for RNA delivery, potentially improving adaptability in soil environments while introducing additional complexity related to microbial survival, interaction with native microbiota, and ecological stability. Collectively, these differences determine the suitability of each approach for specific crop–pathogen systems and influence their scalability and field deployment potential.

In contrast to transgenic HIGS systems, microbial delivery strategies (MIGS) aim to combine environmental adaptability with biological dissemination of RNA signals.

### Microbial Induced Gene Silencing (MIGS)

Microbe-induced gene silencing (MIGS) is an emerging RNAi-based method in which beneficial microbes are engineered or naturally utilized to deliver dsRNA or sRNA that silence essential genes in pathogens (Fang et al. 2024; Luo et al. 2025). Once transferred into the pathogen, these RNAs are processed via the pathogen's RNAi system, causing the degradation of the target messenger RNAs (mRNAs) and inhibition of virulence-related gene expression (Wen et al. 2023; Fang 2024).

Beneficial microbes such as *Trichoderma harzianum* colonize the rhizosphere and antagonize soil-borne pathogens including *V. dahliae* and *Fusarium oxysporum* (Malmierca et al. 2015). Engineered strains can produce sRNAs that cross kingdom boundaries and silence pathogen virulence genes, providing experimental evidence supporting the feasibility of microbe-mediated CK-RNA transfer and gene silencing (Wen et al. 2023). A proof-of-concept study demonstrated successful GFP silencing in *V. dahliae*, confirming that microbe-derived sRNAs can enter fungal pathogens and induce gene silencing (Chen et al. 2025b). By exploiting natural plant–microbe–pathogen interactions, MIGS enables targeted suppression of pathogen genes with minimal non-target effects, reducing reliance on chemical pesticides and supporting integration into sustainable crop management programs (Felippes et al. 2022; Wen et al. 2023).

Beneficial bacteria such as *Bacillus subtilis* and *Pseudomonas putida* can deliver dsRNAs to fungal pathogens through extracellular vesicles, demonstrating bacterial-to-fungal CK-RNA transfer. Application of dsRNA-producing bacteria or their vesicles suppressed *B. cinerea* and *V. dahliae* infections in Arabidopsis and tomato, highlighting the potential of MIGS as a scalable RNA-based crop protection strategy (Niño-Sánchez et al. 2026).

Further optimization is needed to enhance microbial RNA production, stability, and delivery efficiency. Further research may focus on the development of microbial consortia with synergistic capabilities (Gómez-Lama Cabanás and Mercado-Blanco 2025) or incorporation of MIGS with nanocarriers to stabilize dsRNA or sRNA in soil environment (Liu et al. 2024). Ecological safety assessment remains essential to ensure that introduced microbial strains and their RNA products do not disrupt non – target organisms or soil biodiversity (Vatanparast et al. 2024; Dalakouras et al. 2024). Integrating MIGS with cultural practices such as crop rotation, organic amendments, and genetic resistance host breeding may provide an equitable approach to sustainable agriculture.

Despite increasing experimental evidence, MIGS remains less extensively validated under field conditions compared to SIGS and HIGS, and further large-scale studies are required to assess its delivery efficiency, ecological stability, and long-term performance in agricultural environments.

SIGS, HIGS, and MIGS represent complementary RNAi-based strategies for sustainable crop protection that differ primarily in their modes of RNA delivery (Chen et al. 2025a, b; Gebremichael et al. 2021; Ghag 2023). While SIGS relies on exogenous RNA application, HIGS utilizes transgenic plant-mediated expression and MIGS employs beneficial microbes as RNA carriers (Sang and Kim 2020; Beernink 2024; Wen et al. 2023). Together, these approaches demonstrate the translational potential of CK-RNA communication for environmentally sustainable disease management.

## Discussion and future directions

RNA pesticides represent a rapidly emerging industry of the twenty-first century, with the number of globally registered approved products rising continuously (Liu and Smagghe 2026; Qiao et al. 2024). Recently, China officially released the first batch of RNA pesticide products targeting Tobacco Mosaic Virus (TMV), which fully demonstrates the broad industrialization prospects (ICAMA 2026) of such pesticides.

Nevertheless, the research and development of RNA pesticides still faces multiple technical obstacles, among which target genes screening serves as the core step determining the success or failure of product development (Qiao et al. 2024). Although a great many genes can act as functional targets for RNA pesticides, endogenous natural sRNAs are promising targets since they fundamentally reduce potential risks including ecological pollution caused by sRNA off-target effects.

Cross-kingdom sRNAs are native signaling molecules that mediate interspecies communication between microorganisms and plants. Their stable and long-term existence in nature suggests potential environmental biosafety, offering a promising strategy to address the issue of off-target risks. Cross-kingdom RNAs naturally traffic between microbes and plants, and their functional properties are analogous to secondary metabolites. Systematic excavation and characterization of these cross-kingdom RNAs can expand the library of candidate targets and supply more efficient and safe functional targets for the research and development of RNA biopesticides.

The development of RNAi-based crop protection strategies originated from the discovery of environmental RNAi, where gene silencing was induced in the nematode *Caenorhabditis elegans* through exposure to externally supplied double-stranded RNA (dsRNA) (Fire et al. 1998; Timmons et al. 2001). Building on this principle, HIGS was first demonstrated by Nowara et al. (2010), who successfully silenced the *Avra10* gene in *Blumeria graminis* through host-expressed RNA molecules, providing evidence for cross-kingdom RNAi between plants and fungal pathogens. Subsequently, SIGS emerged as a non-transgenic alternative. The first successful application of SIGS for plant protection was reported in 2016, when exogenously applied dsRNA sprays conferred resistance against the fungal pathogens *B. cinerea* and *F. graminearum* (Wang et al. 2016; Koch et al. 2016). More recently, Wen et al. (2023) developed MIGS, an RNAi-based crop protection strategy that utilizes the beneficial rhizosphere fungus *T. harzianum* to produce sRNAs capable of silencing essential genes in the fungal pathogens *V. dahliae* and *F. oxysporum*.

These approaches differ substantially in their delivery mechanisms, effectiveness, and practical applications. SIGS offers a rapid, flexible, and non-transgenic strategy that can be readily deployed during disease outbreaks (Mosquera et al. 2025). However, RNA molecules applied externally are highly vulnerable to degradation by ultraviolet radiation, rainfall, and environmental nucleases, limiting their persistence under field conditions. HIGS provides continuous and tissue-specific production of silencing RNAs within the host plant, resulting in durable protection against pests and pathogens. Nevertheless, its application depends on the development of transgenic crops, which face regulatory restrictions and public concerns regarding genetically modified organisms (GMOs). As the most recently developed approach, MIGS employs beneficial microorganisms as biological RNA delivery systems. Engineered microbes may continuously produce RNA molecules at relatively low cost while simultaneously protecting them from environmental degradation. Furthermore, MIGS can integrate naturally into plant–microbe interactions and may reduce off-target effects (Mitter 2017; Wen et al. 2023; Fang et al. 2025). Despite these advantages, challenges related to microbial establishment under field conditions, controlled RNA release, biosafety assessment, and regulatory approval must be addressed before widespread agricultural application.

A conceptual framework for CK-RNA -mediated plant–microbe communication can be organized into a vertically stratified classification system based on the origin of RNA signals and the degree of human intervention. This framework should be interpreted as a conceptual categorization rather than a strict evolutionary or mechanistic continuum. The top layer consists of SIGS, in which synthetically produced RNA molecules are externally applied to plant surfaces and function as transient bioactive agents without requiring genetic modification of either plants or associated microorganisms. The middle layer represents natural CK-RNA communication, in which endogenous sRNAs are exchanged between plants, pathogens, and symbiotic microorganisms within ecological environments, forming naturally evolved regulatory networks that modulate gene expression, immunity, and interspecies interactions. The bottom layer comprises MIGS, an engineered system in which beneficial microorganisms are used or modified to produce and deliver functional sRNAs to target organisms, acting as living delivery platforms that enable sustained RNA production and environmental protection. Together, this top–middle–bottom classification provides a structured framework to compare naturally occurring RNA signaling processes with engineered RNA delivery technologies and externally applied RNA-based interventions, thereby clarifying their mechanistic relationships and translational roles in future sustainable crop protection.

Overall, by utilizing CK-RNAs—which are naturally filtered through millions of years of plant–microbe co-evolution—as the data foundation for target selection, researchers can identify ultra-specific, environmentally compatible sequences. This inherently reduces the long-standing scientific and public concerns surrounding off-target mutations and ecological contamination. Concurrently, biosynthesizing and encapsulating these evolutionary optimized CK-RNA sequences within engineered microbial hosts (MIGS) effectively neutralizes the severe techno-economic barriers of traditional methods, such as the rapid environmental degradation of naked dsRNA and the exorbitant costs of in vitro chemical synthesis. By merging the elite ecological safety profiles of natural inter-kingdom communication with the robust, low-cost, and protective transport capabilities of microbial bio-factories, the CK-RNA-powered MIGS framework offers a promising emerging direction. Future research prioritizing this dual-mechanism synergy is highly expected to accelerate the transition of RNA biopesticides from laboratory proof-of-concepts into highly effective, commercially scalable realities for global precision agriculture.

## Data Availability

All data are included in this article.

## Abbreviations

CK-RNA: Cross-kingdom RNA
RNAi: RNA interference
HIGS: Host-Induced Gene Silencing
MIGS: Microbe-Induced Gene Silencing
SIGS: Spray-Induced Gene Silencing
sRNAs: Small RNAs
RISC complex: RNA-induced silencing complex
RNA: Ribonucleic acid
mRNA: Messenger RNA
rRNA: Ribosomal RNA
tRNA: Transfer RNA
siRNAs: Small interfering RNAs
miRNAs: MicroRNAs
lncRNAs: Long non-coding RNAs
AGO: Argonaute proteins
milRNA: Micro-RNA like RNA
PR2: Pathogenesis-related protein 2
NF-Y transcription factors: Nuclear factor Y transcription factors
NLR immune receptors: Nucleotide-binding Leucine-Rich Repeat receptor
MdRLKT1: A receptor-like kinase (RLK) gene in apple
ROS: Reactive Oxygen Species
RDR6: RNA-Dependent RNA Polymerase 6
LtRASGEF: Guanine nucleotide exchange factor involved RAS
EVs: Extracellular vesicles
RH11, RH37: RNA helicase 11 and 37
pks29: Polyketide synthase 29
AMF: Arbuscular mycorrhizal fungi
OMVs: Outer membrane vesicles
OsJMT1: Oryza sativa jasmonic acid carboxyl methyltransferase 1
GFP: Green fluorescent protein
vsiRNAs: Virus derived small interfering RNAs
dsRNA: Double-stranded RNA
TGS: Transcriptional gene silencing
PTGS: Post-transcriptional gene silencing
RBSDV: Rice black-streaked dwarf virus
CMV: Cucumber mosaic virus
FHB: Fusarium head blight
Avs: Artificial vesicles
SsOah1: Oxaloacetate acetylhydrolase 1 from *Sclerotinia sclerotiorum*
SsCyp51: Cytochrome P450 51 from *Sclerotinia sclerotiorum*
GST gene: Glutathione S-transferase
AM: Arbuscular mycorrhizal
IPM: Integrated Pest Management
UV: Ultraviolet
*V. dahliae*: Verticillium dahliae
*T. harzianum*: Trichoderma harzianum
*F. graminearum*: Fusarium graminearum
*F. oxysporum*: Fusarium oxysporum
*B. cinerea*: Botrytis cinerea
*P. infestans*: Phytophthora infestans
*N. benthamiana*: Nicotiana benthamiana
